# Mechanistic insights into non-coding RNAs regulate autophagy in chondrocytes and their contribution to osteoarthritis

**DOI:** 10.3389/fmed.2025.1735826

**Published:** 2026-02-02

**Authors:** Bo Wang, Zhihong Wang

**Affiliations:** College of Sports Science, Hefei Normal University, Hefei, China

**Keywords:** autophagy, non-coding RNAs, osteoarthritis, pathological network, therapeutic targets

## Abstract

Osteoarthritis (OA), a chronic degenerative joint disease, arises from a confluence of factors including aging, mechanical injury, and obesity. Autophagy, a fundamental cellular process involving the degradation and recycling of cellular components, plays a critical role in chondrocyte homeostasis and survival under stress. Non-coding RNAs (ncRNAs), a diverse class of RNA molecules with no protein-coding potential, exert significant influence on gene expression through post-transcriptional and epigenetic mechanisms. Growing evidence suggests a crucial interplay between ncRNAs, autophagy, and OA pathogenesis. This review summarizes the multifaceted role of autophagy in OA chondrocytes and delves into the regulatory mechanisms of ncRNAs on OA-associated autophagy, aiming to elucidate the intricate pathological network underlying OA development and identify novel therapeutic targets.

## 1 Introduction

Osteoarthritis (OA), a chronic degenerative joint disease, is characterized by progressive cartilage loss, synovial inflammation, osteophyte formation, and subchondral bone sclerosis (1, 2). Primarily affecting knees, hips, hands, and spine, OA is a global health burden, affecting over 500 million individuals worldwide and ranking as the fourth leading cause of disability (3). A global survey projected that by 2021, over 22% of adults over 40 would be afflicted with knee OA. OA pathogenesis is multifactorial, encompassing aging, obesity, mechanical injury, and chronic inflammation (4). Cartilage homeostasis, maintained by chondrocytes, is crucial for joint health. Aging significantly impacts this homeostasis through mechanisms such as cellular senescence, leading to SASP production, mitochondrial dysfunction, and increased inflammation (5). These age-related changes, alongside obesity, sex, and joint trauma, contribute to the disruption of cartilage homeostasis and the development of OA (6). Currently, effective treatments for OA are limited, and end-stage disease often necessitates joint replacement surgery. However, artificial joints have a finite lifespan and carry inherent risks, resulting in substantial medical and healthcare costs (7, 8).

Regardless of the initial etiology of OA, epigenetic alterations, including changes in gene expression and transcription factor activity, significantly impact the expression of genes crucial for cartilage homeostasis (9). Non-coding RNAs (ncRNAs), such as microRNAs (miRNAs), long non-coding RNAs (lncRNAs), and circular RNAs (circRNAs), are a diverse class of RNA molecules that regulate gene expression at the post-transcriptional and epigenetic levels (10). These ncRNAs have emerged as key players in various biological processes, including cell proliferation, differentiation, apoptosis, and autophagy. Growing evidence suggests that dysregulation of ncRNA expression profiles contributes significantly to chondrocyte senescence, hypertrophy, apoptosis, and extracellular matrix (ECM) degradation, ultimately driving OA pathogenesis (11). Consequently, ncRNAs hold considerable promise as diagnostic biomarkers and therapeutic targets for OA (12, 13).

Autophagy, an essential cellular process for degrading and recycling intracellular components, plays a crucial role in maintaining cellular homeostasis under diverse stress conditions, including nutrient deprivation, oxidative stress, infection, and hypoxia (14). Age-related decline in autophagic activity, characterized by decreased levels of autophagy markers such as LC3, Beclin1, and ULK1, contributes to the accumulation of cellular debris and impaired cellular function in various tissues, including heart, kidney, bone, cartilage, and skeletal muscle (15). In OA, a chronic degenerative joint disease, autophagy dysfunction plays a critical role. Aging, a major risk factor for OA, is associated with impaired autophagy. Specifically, mTOR, a key negative regulator of autophagy, is upregulated in OA cartilage, inhibiting autophagic flux and exacerbating cartilage degeneration (16–18). Furthermore, studies in mice with ATG5 deficiency have demonstrated that autophagy plays a protective role in cartilage by mitigating OA progression, including fibrosis and proteoglycan loss (19). While these findings highlight the significance of autophagy in OA pathogenesis, the precise molecular mechanisms underlying its role remain to be fully elucidated.

Numerous studies have established a strong association between ncRNAs and autophagy (20). Recognizing the pivotal roles of both ncRNAs and autophagy in OA, this review comprehensively examines the intricate role of autophagy in OA pathogenesis (21). Furthermore, we delve into the regulatory influence of ncRNAs, particularly miRNAs, lncRNAs, and circRNAs, on autophagy. By elucidating the intricate interplay within the ncRNA-autophagy axis, we aim to gain deeper insights into the mechanisms that govern cartilage homeostasis and contribute to the development or inhibition of OA, ultimately paving the way for novel diagnostic and therapeutic strategies.

## 2 OA pathogenesis

**Figure 1:**
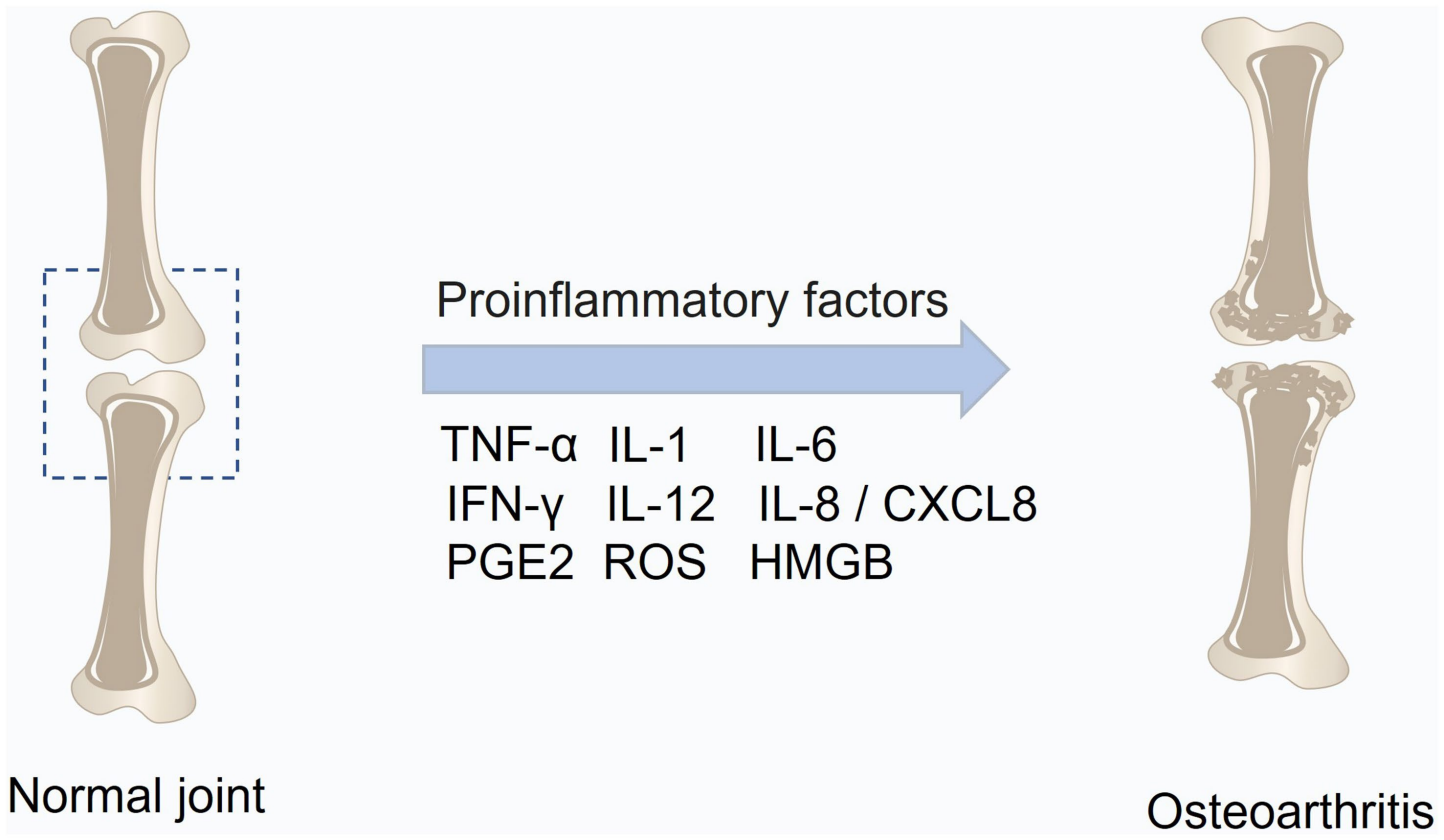
Structural features of a healthy knee joint and pathological alterations in osteoarthritis (OA). A normal knee joint is formed by two articulating bones covered with smooth articular cartilage and enclosed by a capsule lined with synovial tissue. Aging, obesity, and mechanical stress are major contributors to OA onset. These factors trigger elevated production of pro-inflammatory mediators such as IL-1β, IL-6, TNF-α, and NF-κB within cartilage and synovium, suppressing anabolic markers (COL2, ACAN, TGF-β) while enhancing catabolic enzymes (ADAMTS, MMPs) and inflammatory molecules (COX-2, PGE-2). The resulting imbalance leads to progressive cartilage erosion, synovial inflammation, osteophyte formation, and joint degeneration. This figure was generated and edited using Adobe Illustrator (Adobe Inc., United States).

While traditionally viewed as primarily a disease of articular cartilage, OA is now recognized as a multifaceted condition affecting all joint structures, including cartilage, subchondral bone, synovium, and other joint capsule tissues (Figure 1).

### 2.1 Cartilage and chondrocytes

Articular cartilage, composed primarily of water and extracellular matrix (ECM), is synthesized exclusively by chondrocytes through the secretion of collagen type II, aggrecan, and other proteoglycans (22).

In healthy cartilage, chondrocytes exhibit limited mitotic activity, maintaining minimal ECM synthesis (23). This low turnover rate is maintained by the pericellular matrix, which acts as a protective barrier, isolating chondrocytes from the interterritorial matrix and limiting their interaction with ECM components (24). However, in OA, chondrocytes undergo a phenotypic shift. Their repair capacity diminishes, and the delicate balance between matrix synthesis and degradation tilts toward catabolism (25). This is characterized by downregulation of matrix components, including proteoglycans and collagen type II, and upregulation of matrix-degrading enzymes such as MMP13 (26).

In the early stages of OA, elevated matrix synthesis in chondrocytes indicates unsuccessful attempts at repair (27). Damage to the pericellular matrix exposes chondrocytes to surface receptors, including integrins and discoidin structural domain receptors in the interdomain matrix, ultimately resulting in chondrocyte dysfunction (28). Initially, chondrocytes undergo hypertrophic activation, leading to the production of various inflammatory mediators. The actions of these pro-inflammatory factors are crucial in the early stages of OA (29). Interleukin-1 *β* (IL-1β) is a key inflammatory mediator in OA, driving autocrine secretion and the production of other inflammatory cytokines, such as IL-6 and tumor necrosis factor-*α* (TNF-α) (30). The interaction of TNF-α with its receptor, in synergy with IL-1β, activates nuclear factor kappa-B (NF-κB) signaling, promoting the expression of adhesion molecules and further increasing the production of pro-inflammatory mediators (31). As OA progresses, there is a heightened inflammatory response and ongoing secretion of degradative enzymes like ADAMTS5, MMP3, and MMP13, leading to the depletion of proteoglycans and destruction of the collagen network, which contributes to the irreversible nature of OA (32). Chondrocyte apoptosis is a defining event in the final stages of cartilage destruction, exacerbating the catabolism of the cartilage matrix (33). The detachment of cartilage fragments creates a gap between the bone and calcified cartilage, and these free fragments perpetuate the inflammatory cycle, ultimately resulting in lesions in the subchondral bone and synovial tissues (34). This leads to osteosclerosis, thickening of the synovial membrane, and reduced synthesis of synovial molecules, including lubricin and hyaluronic acid.

### 2.2 Subchondral bone

Healthy subchondral bone is composed of two main structures: the subchondral plate and subchondral trabeculae. The subchondral plate is a thin cortical layer situated beneath the calcified cartilage, with a porous region beneath it. The subchondral trabeculae lie beneath the plate and play a crucial role in providing structural support, absorbing mechanical stress, and facilitating the supply of nutrients (35, 36).

Subchondral bone remodeling, characterized by an imbalance between bone formation and resorption driven by aberrant osteoblast–osteoclast coupling, is a hallmark of OA (37). The subchondral bone microarchitecture undergoes significant changes throughout OA progression. Early-stage OA is characterized by increased bone turnover, with elevated bone resorption leading to decreased bone mass (38). This manifests as thinning and increased porosity of the subchondral bone plate, along with reduced trabecular number and thickness. In contrast, advanced OA is characterized by a shift toward increased bone formation, resulting in subchondral bone sclerosis (39). This is evidenced by thickening of the subchondral plate, increased trabecular number and thickness, and decreased trabecular spacing (40).

Despite increased subchondral bone volume, alterations in bone composition, such as an elevated carbonate apatite-to-hydroxyapatite ratio and decreased collagen content, compromise bone mineralization and reduce its elastic modulus (41). This weakens the subchondral bone, diminishing its ability to cushion stress, thereby subjecting it to increased mechanical loading. This vicious cycle of increased stress and bone remodeling exacerbates the progression of OA (42). Concomitant with these changes, fibrovascular invasion through micro-fissures in the subchondral bone disrupts the bone-cartilage interface, facilitating abnormal communication between these tissues (43). This aberrant crosstalk accelerates cartilage degeneration, leading to upward displacement of the tidemark and further aggravating subchondral bone loading and remodeling.

### 2.3 Synovial tissue

The synovium, a specialized connective tissue lining the joint cavity, comprises two layers: an intimal layer containing fibroblast-like and macrophage-like synoviocytes, and a subintimal layer consisting of fibrous connective tissue, blood vessels, and a limited number of immune cells (44). This critical structure plays a vital role in maintaining joint homeostasis. Fibroblast-like synoviocytes are responsible for producing essential components of synovial fluid, including hyaluronic acid and lubricin, which nourish the avascular articular cartilage and facilitate smooth joint movement. Macrophage-like synoviocytes contribute to joint health by clearing debris and combating infections within the synovial fluid (45).

OA synovium exhibits characteristic pathological features, including hyperplasia, fibrosis, and increased vascularity. This inflammatory environment is characterized by significant infiltration of immune cells, such as T cells, B cells, and macrophages, contributing to the formation of a dense inflammatory mass that further degrades articular cartilage and subchondral bone (46). Synovial hyperplasia, driven by excessive production of pro-inflammatory cytokines like TNF-*α* and IL-1, is characterized by increased fibroblast-like synoviocyte proliferation and inhibited apoptosis. Furthermore, these cells contribute to inflammation by phagocytosing cartilage wear particles and releasing inflammatory mediators such as IL-6, nitric oxide, and prostaglandin E2 (47). Pathological vascular proliferation within the synovium exacerbates inflammation. Macrophage-like cells and fibroblasts contribute to this process by producing vascular endothelial growth factor (VEGF), which increases endothelial permeability and promotes plasma extravasation (48).

## 3 The definition and process of autophagy

### 3.1 Definition of autophagy

The term “autophagy” is derived from the Greek words “auto,” meaning self, and “phagy,” meaning to eat or consume (49). Autophagy is a fundamental process in eukaryotic evolution in which a portion of the cytoplasm is enclosed by a double-membrane vesicle known as the autophagosome, which is then transported to the lysosome for degradation (50). Initially, autophagy was viewed primarily as a cellular waste disposal mechanism, but over time, it became clear that constitutive autophagy plays a critical role in eliminating damaged organelles, misfolded or long-lived proteins, and other cellular debris, while also maintaining basal energy homeostasis (51). In contrast, adaptive autophagy mobilizes intracellular components to meet energy demands during various stress conditions such as nutrient deprivation, hypoxia, and other forms of cellular stress (52). This adaptive form of autophagy may have evolved from an ancient survival mechanism in primitive unicellular organisms, enabling them to provide nutrients during energy shortages (53). Overall, autophagy is a key metabolic process that regulates the energy balance within organisms.

### 3.2 Process of autophagy

**Figure 2:**
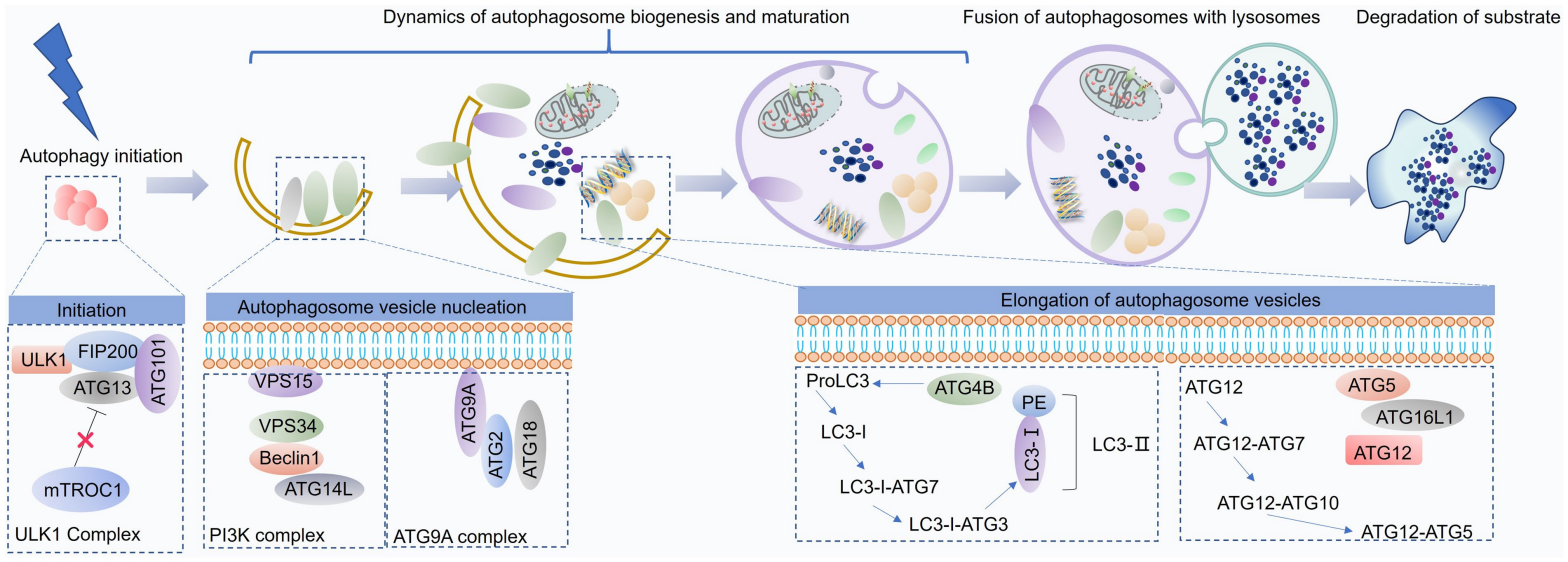
Overview of the autophagy process. Autophagy is a dynamic catabolic pathway comprising several sequential stages: initiation, formation of a double-membrane isolation vesicle, elongation and maturation of the autophagosome, its fusion with lysosomes, and subsequent degradation of the enclosed components. This figure was generated and edited using Adobe Illustrator (Adobe Inc., United States).

As a cellular response to stress, autophagy proceeds through distinct stages: initiation, nucleation, elongation, fusion, and degradation of cytoplasmic components within autophagosomes (Figure 2).

The ULK1 complex, comprising ULK1, ATG13, FIP200, and ATG101, recruits the Beclin1-Vps34 complex (consisting of Beclin1, Vps34, Vps15, and ATG14L) to the sites of autophagosome formation. This recruitment is facilitated by Ambra1, which, upon phosphorylation by ULK1, binds to TRAF6, acting as an E3 ubiquitin ligase to stabilize ULK1 through K63-linked ubiquitination (54). Furthermore, ULK1 directly phosphorylates Beclin1, enhancing the activity of the Vps34 complex (55). The combined activity of these complexes leads to the accumulation of phosphatidylinositol 3-phosphate (PI3P) at the nascent autophagosome membrane. This PI3P enrichment subsequently recruits key autophagy proteins, such as WD-repeat domain phosphoinositide-interacting protein (WIPI) and DFCP1, driving autophagosome formation and expansion (56).

Autophagosome elongation and maturation are facilitated by two ubiquitin-like conjugation systems: the ATG12-ATG5-ATG16L complex and the LC3-phosphatidylethanolamine (PE) conjugation system (57). The ATG12-ATG5-ATG16L complex forms through the conjugation of ATG12 to ATG5, catalyzed by ATG7 and ATG10. This complex then promotes the lipidation of LC3, a key step in autophagosome membrane expansion (58). LC3 undergoes proteolytic cleavage by ATG4 to generate LC3-I, which is subsequently conjugated to PE by the concerted action of ATG7, ATG3, and the ATG12-ATG5-ATG16L complex. This lipidated form of LC3, LC3-II, associates with the autophagosome membrane, driving its maturation (59).

Following their formation, autophagosomes undergo microtubule-dependent trafficking toward lysosomes, where they subsequently fuse. This fusion process, orchestrated by the Rab-SNARE protein machinery and other autophagy-related proteins, facilitates the delivery of autophagosomal cargo to the lysosomal lumen for degradation by lysosomal hydrolases (60).

## 4 Autophagy in OA

### 4.1 Autophagy in cartilage and chondrocyte

**Figure 3:**
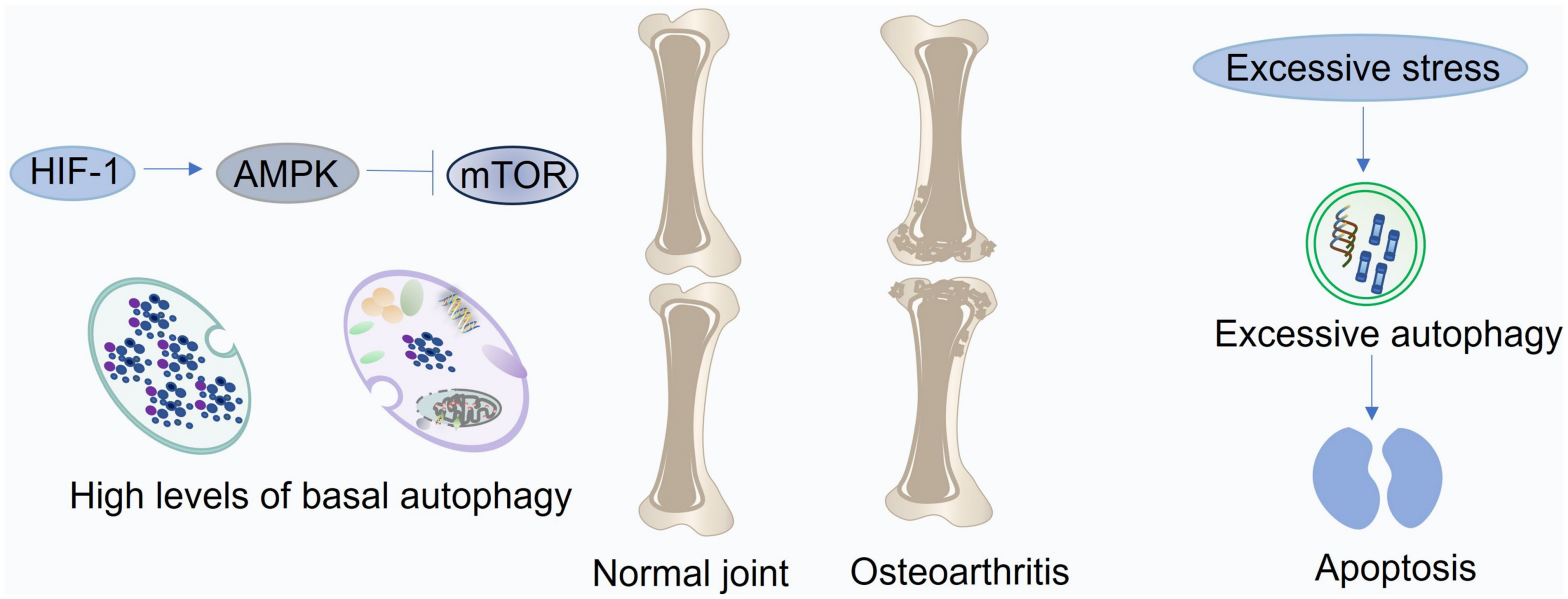
Roles of autophagy in cartilage and chondrocytes. In healthy articular cartilage, autophagy is primarily regulated through the HIF-1/AMPK/mTOR pathway to maintain chondrocyte homeostasis under hypoxic conditions. During the early phase of osteoarthritis (OA), autophagy is transiently enhanced in response to oxidative stress triggered by aging or mechanical injury. As OA progresses, persistent oxidative and inflammatory stress surpasses the compensatory capacity of autophagy, leading to its suppression and resulting in disrupted balance between cartilage anabolism and catabolism. Moreover, excessive autophagy induced by prolonged stress can aggravate chondrocyte apoptosis and cartilage degeneration. This figure was generated and edited using Adobe Illustrator (Adobe Inc., United States).

Chondrocyte function is critical for maintaining cartilage integrity as they synthesize and remodel the ECM (61). However, chondrocytes are susceptible to various stressors, including aging and mechanical injury, leading to inflammation, oxidative stress, and ultimately, disruptions in cartilage homeostasis and the development of OA. Autophagy, a fundamental cellular process for maintaining homeostasis, plays a crucial role in regulating chondrocyte survival and function (Figure 3) (62). While essential for cellular health, autophagy can also have detrimental effects. Excessive autophagy has been implicated in chondrocyte damage and death (63). This highlights the dual role of autophagy in chondrocyte function, where a delicate balance is crucial for maintaining cartilage health. In the following sections, we will delve deeper into the multifaceted role of autophagy in chondrocytes and its implications for OA pathogenesis (64).

#### 4.1.1 Protective autophagy in cartilage and chondrocyte

##### 4.1.1.1 Autophagy and hypoxia

Articular cartilage, an avascular tissue, relies on diffusion for nutrient and oxygen supply from the subchondral bone and synovial fluid (65). This results in a steep oxygen gradient within the cartilage, with oxygen concentrations ranging from approximately 6% at the surface to 1% in the deeper layers. Chondrocytes, adapted to this hypoxic environment, exhibit a strong dependence on hypoxia-inducible factors (HIFs) for their survival and function. HIF-1, expressed in both normal and OA cartilage, promotes chondrocyte phenotype and viability, stimulates matrix synthesis, and supports metabolic adaptation to hypoxia (66). In contrast, HIF-2, predominantly expressed in highly differentiated chondrocytes, drives catabolic processes, including the upregulation of MMPs, ADAMTSs, nitric oxide synthase-2, and prostaglandin-endoperoxide synthase-2, ultimately contributing to cartilage degradation (67). Furthermore, chondrocytes in the hypoxic cartilage environment exhibit increased basal autophagy, which is essential for maintaining cellular homeostasis and promoting collagen synthesis. HIF-1 positively regulates autophagy, while HIF-2 exerts an inhibitory effect (68). This interplay between HIFs and autophagy highlights their crucial roles in chondrocyte function and cartilage health. Conversely, HIF-1 silencing impairs cellular autophagy, leading to MMP13 accumulation and mitochondrial damage (69). The HIF-1-AMPK-mTOR axis serves as a key regulator of autophagy in chondrocytes under hypoxic conditions. Hypoxia stimulates HIF-1 activity, which in turn enhances glycolysis and activates AMPK, leading to subsequent mTOR inhibition and autophagy initiation. This coordinated response ensures chondrocyte viability and facilitates cell cycle progression within the hypoxic environment (70). Interestingly, HIF-2 knockdown elicits a pronounced increase in autophagy in chondrocytes of mouse growth plates. This elevated autophagy is associated with decreased activity of the Akt–mTOR pathway and reduced Bcl-2 expression (71).

##### 4.1.1.2 Autophagy and aging

Autophagy plays a crucial role in chondrocyte differentiation, promoting growth plate maturation and hypertrophy. While robust autophagy is observed in the superficial zone of healthy adult cartilage, its activity declines in aged cartilage (72). Interestingly, autophagy may initially serve as a protective mechanism against early degenerative changes in OA, as evidenced by transient increases in ULK1, Beclin1, and LC3 expression. However, as OA progresses, autophagy activity diminishes, particularly in the superficial zone (73). In advanced stages, autophagy defects become evident across the entire cartilage layer, ultimately contributing to chondrocyte death (74). These findings suggest an age-dependent decline in autophagic activity, paralleling chondrocyte degeneration and preceding cartilage damage (75). Indeed, ATG5-deficient mice exhibit premature chondrocyte senescence and accelerated OA development, potentially linked to cysteoaspartase-dependent apoptosis. In young GFP-LC3 transgenic mice, LC3 and ATG5 expression was most prominent in superficial and middle zone chondrocytes (76). Conversely, senile mice exhibited a significant reduction in autophagosome numbers within chondrocytes, alongside a marked decrease in vesicle abundance in the subchondral bone. Concomitantly, impaired autophagy exacerbated OA-like changes in the synovium (77). This evidence suggests that autophagy defects contribute to early senescent changes in cartilage, ultimately leading to aberrant matrix synthesis, catabolic gene expression, and ECM breakdown. Furthermore, a recent study reported that fibroblast-like synoviocyte senescence, characterized by impaired autophagy and upregulated SASP factors, exacerbates OA progression (78). Notably, restoring autophagy in these senescent cells reversed the senescence phenotype, suppressed SASP secretion, and mitigated cartilage destruction by inhibiting GATA4 (79).

##### 4.1.1.3 Autophagy and mechanical injury

Acute or prolonged abnormal mechanical loading compromises chondrocyte regenerative capacity, contributing to the development of degenerative lesions and the onset of OA (80). Mechanical stress triggers the activation of multiple inflammatory and oxidative stress pathways, driving joint inflammation through the upregulation of cartilage-degrading enzymes, ultimately leading to chondrocyte apoptosis, ECM degradation, and aberrant subchondral bone remodeling (81). Experimental studies have demonstrated that mechanical injury inhibits autophagy. For instance, 500 ms of mechanical shock induced chondrocyte apoptosis and sulfated glycosaminoglycan loss in a time-dependent manner, accompanied by a significant decrease in ULK1, Beclin1, and LC3 expression in the superficial cartilage at 48 h post-injury (82). Notably, exogenous autophagy activation mitigated the chondrocyte damage induced by mechanical injury (83).

Conversely, appropriate mechanical loading exerts beneficial effects on chondrocytes, alleviating senescence and inflammation, regulating autophagy, and promoting cartilage repair (84). In a rat model of DMM, treadmill exercise inhibited excessive autophagy by activating the PI3K-Akt–mTOR pathway, thereby mitigating cartilage inflammation and degeneration (85). Furthermore, in another mouse OA model, appropriate mechanical loading enhanced autophagy levels in cartilage, as evidenced by increased LC3 expression and decreased p62 levels, ultimately promoting chondrocyte regeneration and facilitating cartilage repair (86).

##### 4.1.1.4 Autophagy and inflammation

In OA, chondrocytes encounter a milieu of stressors, including mechanical injury, cellular senescence, and exposure to inflammatory cytokines and degradation products (87). These stressors activate various signaling pathways, leading to aberrant gene expression, including increased production of pro-inflammatory cytokines such as TNF-*α*, IL-1β, and IL-6. These cytokines, in turn, stimulate the expression of MMPs and ADAMTSs, driving cartilage matrix degradation (88). Chondrocytes themselves contribute to the inflammatory milieu by expressing receptors for various pro-inflammatory factors and chemokines, acting as both sources and targets of inflammatory mediators within the joint (89). Furthermore, inflammation within the synovium and subchondral bone significantly contributes to cartilage degeneration in OA (90). These inflammatory factors have been shown to exacerbate OA by inhibiting autophagy and promoting chondrocyte senescence (91). IL-1β-induced inflammation in chondrocytes significantly activates the PI3K-Akt–mTOR signaling pathway, inhibiting autophagy and promoting cell cycle arrest, characterized by G1 phase blockade and reduced S phase progression (92). Conversely, inhibiting the PI3K-Akt–mTOR pathway enhances autophagy and attenuates inflammation in chondrocytes from OA rats. Furthermore, PPARγ deficiency leads to increased expression of inflammatory markers, such as inducible nitric oxide synthase and cyclooxygenase-2, accompanied by elevated mTOR expression and suppressed autophagy. This exacerbates inflammation within the cartilage and drives OA progression (93). The mTOR pathway also plays a crucial role in regulating synovitis. Inhibition of mTOR activates autophagy, suppressing osteoclastogenesis and IL-1 expression in the synovium, thereby protecting cartilage from erosion (94). Additionally, the MAPK/NF-κB pathway contributes to the suppression of autophagy in OA. Recent studies have highlighted the involvement of epigenetic modifications in regulating autophagy and inflammation in OA (95). Specifically, methyltransferase-like 3 stabilizes Bcl-2 mRNA through the m6A/Yth m6A RNA-binding protein 1 axis, thereby inhibiting Beclin1-mediated autophagy and exacerbating chondrocyte apoptosis and subchondral bone degeneration *in vivo* (96).

##### 4.1.1.5 Autophagy and oxidative stress

Oxidative stress arises from an imbalance between reactive oxygen species (ROS) production and antioxidant defenses within the mitochondria (95). In OA, this imbalance is exacerbated by a disruption in redox homeostasis, particularly involving superoxide dismutase 2 (97). In OA chondrocytes, stimuli such as mechanical injury and senescence activate cytokine receptors and toll-like receptors (TLRs), leading to increased mitochondrial ROS production and decreased expression of antioxidant enzymes, including superoxide dismutase 1 and 2 (98, 99). This oxidative environment promotes the modification of intracellular and extracellular components, inhibits collagen synthesis, and activates MMPs and ADAMTSs, driving cartilage degradation (100). Furthermore, degradation products stimulate the activation of NADPH oxidase and inducible nitric oxide synthase, creating a vicious cycle of inflammation and ROS production, further exacerbating joint degeneration (101).

Autophagy exhibits a biphasic response in OA. While elevated in the early stages, likely as a protective mechanism against oxidative stress, autophagy activity declines in the mid-late stages of the disease (102). The accumulation of p62, a marker of impaired autophagy, in OA chondrocytes exposed to oxidative stress promotes chondrocyte apoptosis. Alginate treatment effectively restores autophagic flux by targeting BNIP3, a key regulator of autophagy, thereby mitigating oxidative stress-induced damage (103). Furthermore, TGFβ1 pretreatment enhances LC3-II expression in H2O2-treated chondrocytes via forkhead box O1, mitigating oxidative stress-induced cell death (104). Additionally, glabridin, a natural antioxidant, inhibits mTOR signaling, thereby activating autophagy and protecting chondrocytes from oxidative damage (105).

#### 4.1.2 Detrimental autophagy in cartilage and chondrocyte

These findings mentioned above suggest that autophagy plays a crucial but complex role in OA pathogenesis. While impaired autophagy contributes to OA progression in aging and disease models, restoring appropriate autophagic flux can mitigate inflammation, oxidative stress, and other pathological hallmarks of OA. However, excessive autophagy activation can also have detrimental effects, highlighting the need for a delicate balance in autophagy activity for optimal cartilage health (106).

Mitofusin 2, a key regulator of autophagy and inflammation, significantly disrupts autophagic homeostasis when overexpressed. This overexpression inhibits the PI3K/AKT/mTOR pathway, leading to excessive autophagy, increased ROS production, and subsequent inflammation and cartilage degeneration (107). While Lee et al. (108) observed increased autophagy and autophagic cell death in OA chondrocytes, characterized by elevated LC3 and Beclin-1 expression in the mid-upper zone, Caramés et al. (109) reported decreased expression of these autophagy markers in the superficial zone of OA cartilage, potentially due to chondrocyte loss in this region. However, prolonged exposure to acidic conditions (pH 6.0 for 48 h) activates Acid-sensitive ion channel 1a, inducing chondrocyte senescence through sustained autophagy activation (110). This highlights that excessive autophagy can have detrimental effects, contributing to cellular dysfunction. Oxidative DNA damage can also disrupt autophagic flux. In response to acute oxidative stress, healthy chondrocytes exhibit a transient increase in autophagy activity, followed by a return to basal levels within 24 h. In contrast, OA chondrocytes exhibit sustained elevated autophagy levels, likely due to mitochondrial dysfunction and increased ROS production (111). This persistent oxidative stress in OA chondrocytes leads to the formation of a pre-autophagic pool, impairing the ability of these cells to appropriately modulate autophagy in response to further oxidative stress. This dysregulated autophagy can ultimately drive chondrocyte death (112).

Autophagy plays a multifaceted role in OA, responding to a variety of stressors, including aging, hypoxia, mechanical loading, inflammation, and oxidative stress. While autophagy can initially serve as a protective mechanism against these stressors, mitigating cellular damage, excessive or prolonged activation can have detrimental consequences (113). For example, mitochondrial dysfunction induced by oxidative stress can lead to spatiotemporal dysregulation of autophagy, resulting in excessive autophagic flux and ultimately, chondrocyte death (114). Similarly, prolonged exposure to stress can overwhelm cellular compensatory mechanisms, leading to sustained high levels of autophagy and subsequent cell death. These findings underscore the complex and dynamic nature of autophagy in OA, highlighting the critical importance of maintaining a delicate balance between beneficial and detrimental autophagic activity (115). The precise impact of autophagy likely depends on the type, intensity, and duration of the stress stimulus. While research has implicated autophagy in OA pathogenesis, further investigation is needed to define the precise threshold between beneficial and detrimental autophagy in chondrocytes under various pathological conditions (116). Despite these limitations, autophagy holds significant promise as a therapeutic target for OA. However, therapeutic interventions should be carefully designed to modulate autophagy activity within a therapeutic window, avoiding both insufficient and excessive autophagy (117).

### 4.2 Autophagy in subchondral bone

Subchondral bone remodeling plays a critical role in OA pathogenesis. Early OA is characterized by increased bone resorption, leading to decreased bone volume. In contrast, late-stage OA is characterized by excessive bone formation and sclerosis. These alterations in bone metabolism highlight the potential of targeting key signaling molecules in osteoblasts and osteoclasts as therapeutic strategies for OA (118). Autophagy plays a crucial role in bone metabolism, influencing osteoblast activity, differentiation, and mineralization. Similarly, autophagy regulates osteoclast differentiation and maturation (119). The RANK/RANKL/OPG signaling pathway governs subchondral bone remodeling. In DMM-induced OA, decreased bone volume and mineral density are associated with increased RANKL expression and enhanced osteoclast formation. Notably, rapamycin-induced autophagy activation inhibits osteoclastogenesis through the RANK/RANKL/OPG axis, suggesting a potential therapeutic avenue for OA treatment (120). Early OA is characterized by alterations in subchondral bone structure, including changes in osteoblast phenotype and mineralization. Defective autophagy contributes to impaired osteoblast mineralization in early OA, while enhanced autophagy restores osteoblast function and alleviates subchondral bone thinning (121). In late-stage OA, 15-lipoxygenase-1, a pro-inflammatory lipid mediator, is upregulated in subchondral osteoblasts. This upregulation inhibits autophagy through mTORC1 activation, while simultaneously stimulating osteoblast activity, leading to increased expression of osteoblastic markers such as RUNX2, COL1, and OCN. This aberrant osteoblast activity contributes to abnormal bone remodeling and exacerbates OA (122). Furthermore, 15-lipoxygenase-1-mediated mTORC1 activation promotes AMPK phosphorylation, further suppressing autophagy and stimulating TGF-β1 expression, which enhances osteoblast proliferation and differentiation, ultimately contributing to abnormal ECM synthesis and mineralization within the subchondral bone. While these findings highlight the crucial role of autophagy in regulating subchondral bone remodeling in OA, further research is needed to fully elucidate the mechanisms underlying autophagy regulation of osteoblast and osteoclast function in this context (122).

### 4.3 Autophagy in synovium

Synovial inflammation plays a critical role in OA pathogenesis. With aging, fibroblast-like synoviocytes (FLS) undergo senescence, characterized by increased secretion of inflammatory cytokines, MMPs, and senescence-associated secretory proteins (SASPs), thereby contributing to cartilage destruction (123). Previous studies have demonstrated that FLS senescence in OA is closely associated with impaired autophagy. Restoring autophagic flux effectively mitigates FLS senescence and SASP secretion, suggesting a crucial role for autophagy in attenuating OA progression. Interestingly, Delco et al. (124) reported that excessive m6A RNA methylation, mediated by the methyltransferase METTL3, inhibits autophagy in OA-FLS by destabilizing ATG7 mRNA. Silencing METTL3 enhances autophagic flux, suppresses GATA4 expression, and attenuates FLS senescence, demonstrating a therapeutic potential for targeting m6A methylation in OA (125). While these findings highlight the importance of autophagy in regulating synovial inflammation, further research is needed to fully elucidate the underlying mechanisms and explore potential therapeutic strategies (126).

## 5 The mechanism of ncRNA-mediated autophagy in OA

Numerous studies have highlighted the intricate interplay between ncRNAs and autophagy in age-related diseases, including OA (127). These ncRNAs can be broadly classified based on their ability to either promote or inhibit autophagic flux. While the pivotal roles of both autophagy and ncRNAs in OA pathogenesis are well-established, the precise molecular mechanisms underlying their crosstalk remain largely unexplored (128). Further research is warranted to elucidate the intricate relationship between ncRNA dysregulation and autophagy deficiency in the context of OA.

### 5.1 MiRNAs and autophagy in OA

**Figure 4:**
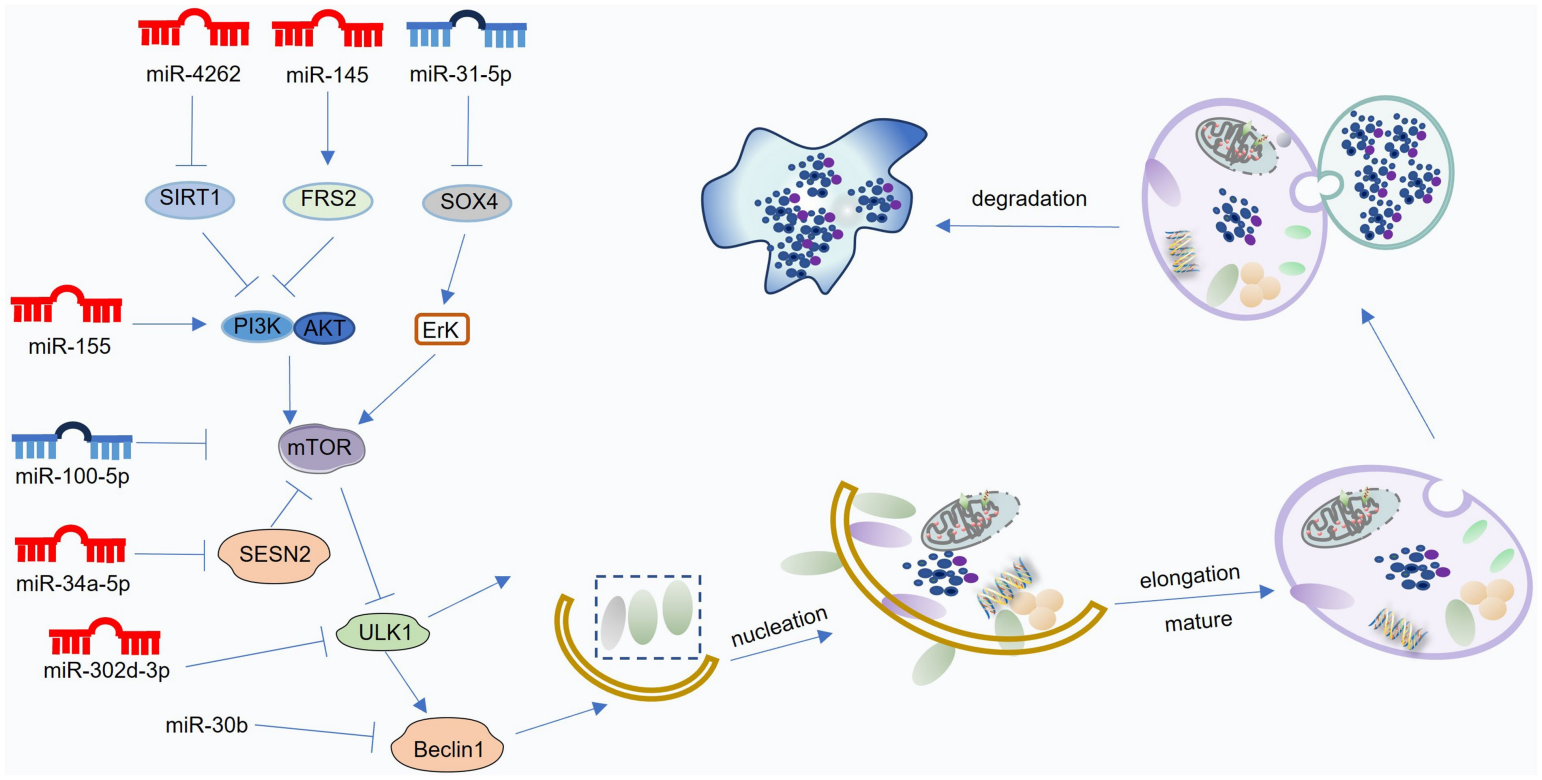
Mechanisms of miRNA-mediated regulation of autophagy in osteoarthritis (OA). MiRNAs modulate various stages of the autophagic process—including initiation, vesicle formation and elongation, autolysosome maturation, and degradation—by targeting key regulators such as mTOR, Beclin1, ATG proteins, and LC3. Blue miRNAs denote those that enhance autophagy and protect against OA progression, whereas red miRNAs indicate those that suppress autophagy and contribute to OA development. This figure was generated and edited using Adobe Illustrator (Adobe Inc., United States).

MiRNAs are small, single-stranded RNA molecules that regulate gene expression by binding to the 3′ untranslated regions (UTRs) of target mRNAs (129). This regulatory network is complex, with each miRNA capable of targeting multiple genes and multiple miRNAs potentially originating from a single mRNA transcript. MiRNAs exert profound influence on diverse cellular processes, including development, proliferation, differentiation, cell cycle regulation, and autophagy (130). Numerous studies have demonstrated the ability of miRNAs to directly regulate autophagy at various stages, including initiation, vesicle formation, and autophagosome-lysosome fusion. Furthermore, miRNAs can indirectly modulate autophagy by targeting upstream signaling pathways, such as the mTOR, sirtuin, and AMPK pathways (131). The following sections will discuss the role and mechanisms of miRNA-mediated autophagy regulation in OA (Figure 4; Table 1).

**Table 1:** MiRNAs mediated autophagy on OA.

Model	MicroRNA expression	Targeting pathway	Effect on autophagy	Effect on cartilage or chondrocyte	Ref.
Human OA chondrocytes	miR-335–5p ↓	mTOR ↓	Promote	Inflammation ↓ cell viability ↑	(203)
Human OA/C20A4 cell	miR-145 ↓	FRS2 PI3K-Akt–mTOR ↓	Promote	Promote	(204)
OA model IL-1β stimulated chondrocytes	miR-31–5p ↓	SOX4 ↓ ERK-mTORC1 ↓	Promote	proliferation↑ apoptosis ↓	(205)
Hypoxia-induced chondrocytes	miR-146a ↑	Bcl-2 ↓	Promote	ECM degeneration ↓	(206)
Hypoxia-induced primary chondrocytes	miR-146a ↑	Traf6-IRAK1-Bcl-2↓	Promote	ECM degeneration ↓	(207)
DMM induced OA	miR-17–5p ↓	P62 ↓	Promote	cartilage injury ↓	(208)
IL-1β stimulated primary chondrocytes	miR-766–3p ↓	AIFM1 ↓	Promote	Apoptosis ↓ ECM ↑	(209)
Human primary chondrocytes	miR-140–5p/149 ↓	FUT1 ↓	Promote	Apoptosis ↓	(210)
Human primary chondrocytes	miR-155 ↑	mTOR ↑	Inhibit	Apoptosis ↑	(211)
Polychlorinated biphenyls reduced OA	miR-155 ↑	RICTOR-Akt- mTOR ↑	Inhibit	cartilage matrix ↓	(212)
Developmental dysplasia of the hip reduced OA	miR-34a-5p ↑	SESN2 ↓, mTOR ↑	Inhibit	Proliferation ↓ cell migration ↓	(213)
TNF-α stimulated chondrocytes	miR-4262 ↑	SIRT1 ↓	Inhibit	Apoptosis ↑	(214)
CHON-001 cell	miR-302d-3p ↑	ULK1 ↓	Inhibit	Inflammation ↑	(215)
DMM-induced OA	miR-375 ↑	ATG2b ↓	Inhibit	Apoptosis ↑	(216)
TNF-α induced ATDC5 cells	miR-30b ↑	BECN1, ATG5 ↓	Inhibit	Apoptosis ↑	(217)
chondrocytes	miR-20a ↑	ATG7 ↓	Inhibit	chondrogenic differentiation ↓	(218)
chondrocytes	miR-20a ↑	ATG7 ↓	Inhibit	chondrogenic differentiation ↓	(219)
Human OA chondrocytes	miR-20 ↑	ATG10 ↓	Inhibit	Proliferation ↓	(220)
ACLT induced OA	miR-128a ↑	ATG12 ↓	Inhibit	Apoptosis ↑ ECM degradation ↑	(221)
miR-378 transgenic (TG) mice	miR-378 ↑	ATG2a ↓	Inhibit	BMSCs chondrogenesis ↓	(222)
Dexamethasone reduced OA	miR-1912–3p↑	CTSD ↓	Inhibit	MMP3, MMP13, ADAMTS5 ↑ cartilage matrix ↓	(223)
ACLT induced OA	miR-146a-5p ↑	NUMB ↓	Inhibit	Apoptosis ↑	(224)

#### 5.1.1 Positive miRNAs to suppress OA pathogenesis

Emerging evidence suggests that miRNAs can exert protective effects in OA by promoting autophagy. A common mechanism involves targeting the mTOR signaling pathway. Several miRNAs, including miR-335-5p, activate the AMPK-mTOR pathway, leading to mTOR inhibition and subsequent autophagy activation (132). MiR-145 targets FRS2, inhibiting PI3K/Akt/mTOR signaling, thereby promoting autophagy and mitigating oxidative stress (133). MiR-31-5p negatively regulates SOX4, inhibiting mTORC1 activation through the ERK pathway, ultimately enhancing autophagy and promoting chondrocyte survival (134).

Beyond mTOR, other autophagy-related proteins, such as Bcl-2 and p62, are also subject to miRNA regulation. The Bcl-2 pathway plays a crucial role in miRNA-mediated autophagy regulation, particularly under hypoxic conditions. Zhang et al. (135) demonstrated that HIF-1α upregulates miR-146a expression in response to hypoxia. MiR-146a, in turn, inhibits Bcl-2, promoting autophagy and enhancing chondrocyte survival. Furthermore, miR-146a directly targets TRAF6 and IRAK1, key mediators of the NF-κB pathway, thereby attenuating inflammation (136). Another example is miR-145, which targets FRS2, inhibiting PI3K/Akt/mTOR signaling and promoting autophagy. MiR-31-5p, downregulated in OA, targets SOX4. This downregulation relieves the inhibitory effect of SOX4 on mTORC1, promoting autophagy and enhancing chondrocyte survival (137). These findings highlight the intricate interplay between miRNAs, autophagy, and cellular signaling pathways in the context of OA. Hypoxia-induced miR-146a promotes chondrocyte autophagy by inhibiting Bcl-2 expression through the TRAF6-IRAK1 pathway, independent of SMAD-4 (138). P62/SQSTM1, a key autophagy receptor, plays a critical role in autophagosome formation by transporting ubiquitinated proteins to the lysosome for degradation. MiR-17-5p directly targets and downregulates p62 expression. In the DMM-induced OA model, decreased p62 levels correlated with reduced miR-17-5p expression, consistent with its downregulation in IL-1β-treated SW1353 chondrosarcoma cells (139). MiR-17-5p overexpression significantly suppressed p62 expression in SW1353 cells, leading to enhanced autophagic activity. Furthermore, p62 knockdown partially reversed the inhibitory effect of miR-17-5p inhibition on LC3 expression, suggesting a crucial role for p62 in regulating autophagy in response to miR-17-5p modulation (140).

Beyond directly targeting autophagy-related proteins, miRNAs can also regulate autophagy through indirect mechanisms. For example, Wang et al. (141) demonstrated that miR-140-5p/149 directly targets fucosyltransferase 1 (FUT1) in human primary chondrocytes. Overexpression of miR-140-5p/149 inhibits FUT1 expression, leading to enhanced autophagy, increased cell proliferation, and reduced apoptosis. Conversely, FUT1 knockdown rescued the inhibitory effects of miR-140-5p/149 inhibition on autophagy. These findings suggest that the miR-140-5p/149-FUT1 axis plays a crucial role in regulating autophagy and chondrocyte homeostasis, highlighting its potential as a novel therapeutic target for OA.

#### 5.1.2 Negative miRNAs to promote OA pathogenesis

Numerous studies have implicated miRNAs in OA pathogenesis through their ability to inhibit autophagy. MiR-155 targets the PI3K-Akt–mTOR pathway, suppressing autophagy by inhibiting ULK1, FOXO3, ATG14, ATG5, ATG3, and LC3 expression (142). Additionally, miR-155 directly targets RICTOR, a key component of the mTORC2 complex, further activating the Akt/mTOR signaling pathway, leading to impaired autophagy and exacerbated OA (143). MiR-34a-5p targets SESN2, a negative regulator of mTOR signaling. Upregulation of miR-34a-5p in OA cartilage suppresses SESN2 expression, leading to increased mTOR activity and impaired autophagy (144). Conversely, miR-34a-5p knockdown enhances SESN2 expression and autophagy activity. Besides, MiR-4262 targets SIRT1, a key regulator of cellular metabolism. In TNF-*α*-treated chondrocytes, miR-4262 overexpression inhibits SIRT1 expression, leading to increased PI3K/Akt/mTOR signaling and subsequent suppression of autophagy (145). Conversely, SIRT1 overexpression inhibits PI3K/Akt/mTOR signaling, promoting autophagy and mitigating the detrimental effects of miR-4262 overexpression, such as apoptosis and impaired ECM synthesis (146).

MiRNAs can exert detrimental effects in OA by targeting key autophagy proteins. MiR-378 directly targets ATG2a, a crucial protein involved in autophagosome formation. In miR-378 transgenic mice, downregulated ATG2a expression leads to impaired autophagic vesicle formation and contributes to OA development (147). MiR-375 directly targets ATG2b, inhibiting autophagosome membrane closure. This miR-375-mediated inhibition of ATG2b exacerbates endoplasmic reticulum stress and promotes chondrocyte apoptosis (148). MiR-30b directly targets Beclin1 and ATG5, inhibiting autophagy initiation and elongation, respectively. MiR-30b overexpression in chondrocytes promotes apoptosis and inhibits autophagy, likely contributing to OA pathogenesis (149). Additionally, MiR-20a targets ATG7, a key protein involved in LC3 lipidation. MiR-20a overexpression in ATDC-5 cells downregulates ATG7, leading to impaired autophagosome formation and reduced chondrogenesis (150). MiR-128a targets ATG12, inhibiting LC3 lipidation and autophagosome formation. Additionally, miR-128a overexpression is associated with H3K27 hypomethylation and enhancer of zeste homolog 2 inactivation, further contributing to impaired autophagy and exacerbated OA progression (151). These findings demonstrate that miRNAs can regulate autophagy at multiple levels, including initiation, elongation, and maturation, by targeting key autophagy-related proteins such as Beclin1, ATG5, ATG7, and ATG12. Dysregulation of these miRNAs can contribute to OA pathogenesis by disrupting autophagic flux and promoting chondrocyte dysfunction (152).

MiRNAs can indirectly inhibit autophagy by targeting factors that regulate autophagic flux. MiR-1912-3p targets CTSD, a lysosomal protease crucial for autophagic degradation. In a model of fetal dexamethasone exposure, miR-1912-3p downregulates CTSD expression, impairing autophagy and increasing offspring susceptibility to OA (153). Besides, MiR-146a-5p targets NUMB, a negative regulator of autophagy. Upregulation of miR-146a-5p in OA cartilage inhibits NUMB expression, leading to decreased autophagy, increased apoptosis, and exacerbated OA progression (154).

### 5.2 LncRNAs and autophagy in OA

**Figure 5:**
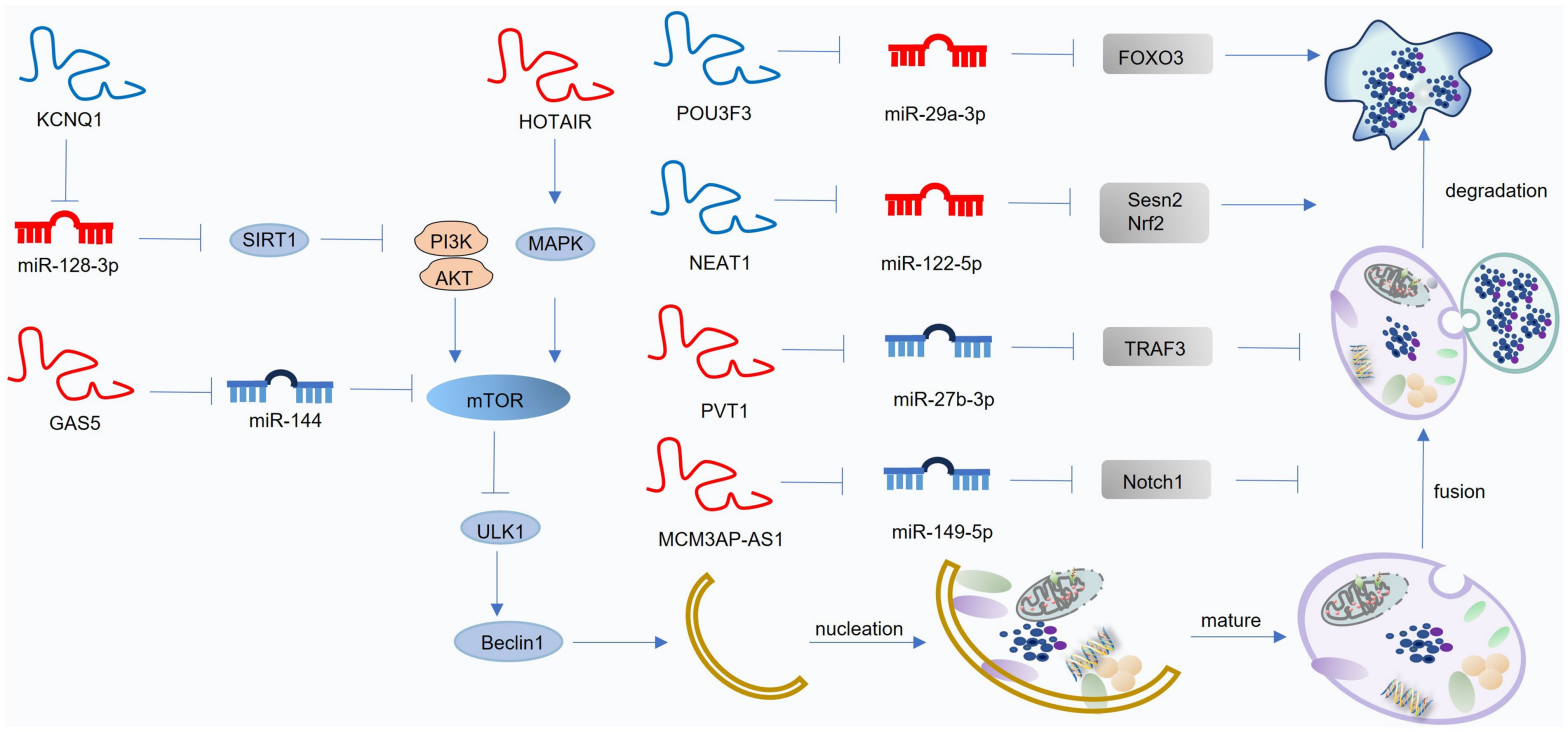
Mechanisms of lncRNA-mediated regulation of autophagy in osteoarthritis (OA). LncRNAs mainly act as competing endogenous RNAs (ceRNAs) that interact with miRNAs to modulate autophagy-related pathways. Blue lncRNAs indicate those that activate autophagy and suppress OA progression, while red lncRNAs denote those that inhibit autophagy and facilitate OA development. This figure was generated and edited using Adobe Illustrator (Adobe Inc., United States).

lncRNAs are non-coding RNA transcripts exceeding 200 nucleotides in length with limited or no protein-coding potential (155). LncRNAs exert diverse regulatory functions, including chromatin modification, mRNA splicing, and translational control. Furthermore, lncRNAs interact with miRNAs through several mechanisms. For example, LncRNAs can act as ceRNAs, competing with mRNAs for miRNA binding, thereby influencing gene expression (156). Besidesm, some lncRNAs serve as precursors for miRNAs, such as npc83 and npc521, which generate miR-869a and miR-160c, respectively (157). LncRNAs can also be direct targets of miRNAs, leading to their degradation. In OA, lncRNAs primarily function as ceRNAs, regulating gene expression by modulating miRNA activity. These lncRNAs influence various cellular processes, including inflammation, proliferation, apoptosis, and autophagy, ultimately impacting cartilage homeostasis (158). Numerous lncRNAs have been implicated in regulating autophagy in OA, either by directly targeting autophagy-related genes or by acting as miRNA sponges (Figure 5; Table 2).

**Table 2:** LncRNAs mediated autophagy on OA.

Model	LncRNA expression	Targeted miRNAs	Targeting pathway	Effect on autophagy	Effect on cartilage or chondrocyte	Ref.
Human OA cartilage	lncRNA POU3F3 ↓	miR-29a-3p	FOXO3 ↑	Promote	inflammation ↓ apoptosis ↓	(225)
Human OA cartilage, DMM induced OA chondrocyte	lncRNA NEAT1 ↓	miR-122–5p	Sesn2, Nrf2 ↑	Promote	apoptosis ↓ proliferation ↑	(226)
HFD induced OA	lncRNA KCNQ1 ↓	miR-128–3p	SIRT1 ↑	Promote	inflammation ↓ apoptosis ↓	(227)
DMM induced OA	lncRNA HOTAIR ↑	/	p38MAPK ↑	Inhibit	inflammation ↑ ECM degeneration ↑	(228)
Human OA cartilage chondrocyte	lncRNA HOTAIR ↑	miR-130a-3p	/	Inhibit	apoptosis ↑	(229)
DMM induced OA	lncRNA GAS5 ↑	miR-144	mTOR ↑	Inhibit	apoptosis ↑	(230)
Human OA cartilage	lncRNA GAS5 ↑	miR-21	LC3B ↓	Inhibit	apoptosis ↑	(231)
Human OA cartilage, IL-1β induced C28/I2	lncRNA PVT1 ↑	miR-27b-3p	TRAF3 ↑	Inhibit	inflammation ↑ apoptosis ↑	(232)
Human OA cartilage	lncRNA	miR-149–5p	Notch1 ↑	Inhibit	ECM degeneration ↑	(233)
Human OA synoviocytes	lncRNA PCGEM1 ↑	miR-770	/	Promote	apoptosis ↓ proliferation ↑	(234)

#### 5.2.1 Positive lncRNAs to suppress OA pathogenesis

Several lncRNAs exert protective effects in OA by promoting autophagy through their miRNA sponging activity. For example, downregulated in OA cartilage, lncRNA POU3F31 acts as a ceRNA for miR-29a-3p, thereby increasing FOXO3 expression and promoting autophagy. This, in turn, inhibits inflammation and apoptosis in chondrocytes (159). Downregulated in HFD-induced OA, lncRNA KCNQ1OT1 acts as a ceRNA for miR-34a-5p. Upregulation of lncRNA KCNQ1OT1, for example, by salbutamol B treatment, inhibits miR-34a-5p activity, leading to increased SIRT1 expression, enhanced autophagy, and reduced inflammation in chondrocytes (160).

#### 5.2.2 Negative lncRNAs to promote OA pathogenesis

Conversely, some lncRNAs contribute to OA progression by inhibiting autophagy. For instance, lncRNA HOTAIR inhibits the p38MAPK pathway, suppressing autophagy and enhancing inflammation (161). Additionally, HOTAIR acts as a ceRNA for miR-130a-3p, further inhibiting autophagy and promoting apoptosis by downregulating miR-130a-3p-mediated suppression of the mTOR pathway (162). Upregulated in OA, lncRNA GAS5 acts as a ceRNA for miR-144. Silencing of lncRNA GAS5 increases miR-144 expression, inhibiting mTOR signaling and enhancing autophagy, ultimately protecting chondrocytes from apoptosis (163). Moreover, LncRNA GAS5 acts as a ceRNA for miR-21, inhibiting its activity. This leads to enhanced autophagy by preventing miR-21-mediated suppression of autophagy complex formation, ultimately mitigating apoptosis and ECM degradation (164). Besides, Upregulated in OA, lncRNA PVT1 acts as a ceRNA for miR-27b-3p, promoting TRAF3 expression and inhibiting autophagy. This contributes to increased inflammation and apoptosis (165). Upregulated by SOX4, lncRNA MCM3AP-AS1 acts as a ceRNA for miR-149-5p, promoting Notch1 expression. This dysregulated axis contributes to impaired autophagy and exacerbated ECM degradation in OA (166).

Beyond chondrocyte dysfunction, synovial inflammation plays a critical role in OA pathogenesis. Activated synoviocytes secrete pro-inflammatory cytokines, such as IL-1β and TNF-*α*, which stimulate the production of matrix metalloproteinases (MMPs) and other degradative enzymes, leading to cartilage destruction (167). LncRNA PCGEM is upregulated in OA synoviocytes. By acting as a ceRNA for miR-770, lncRNA PCGEM promotes autophagy, inhibits apoptosis, and stimulates proliferation in synoviocytes, thereby exacerbating OA pathology (168).

### 5.3 CircRNAs and autophagy in OA

**Figure 6:**
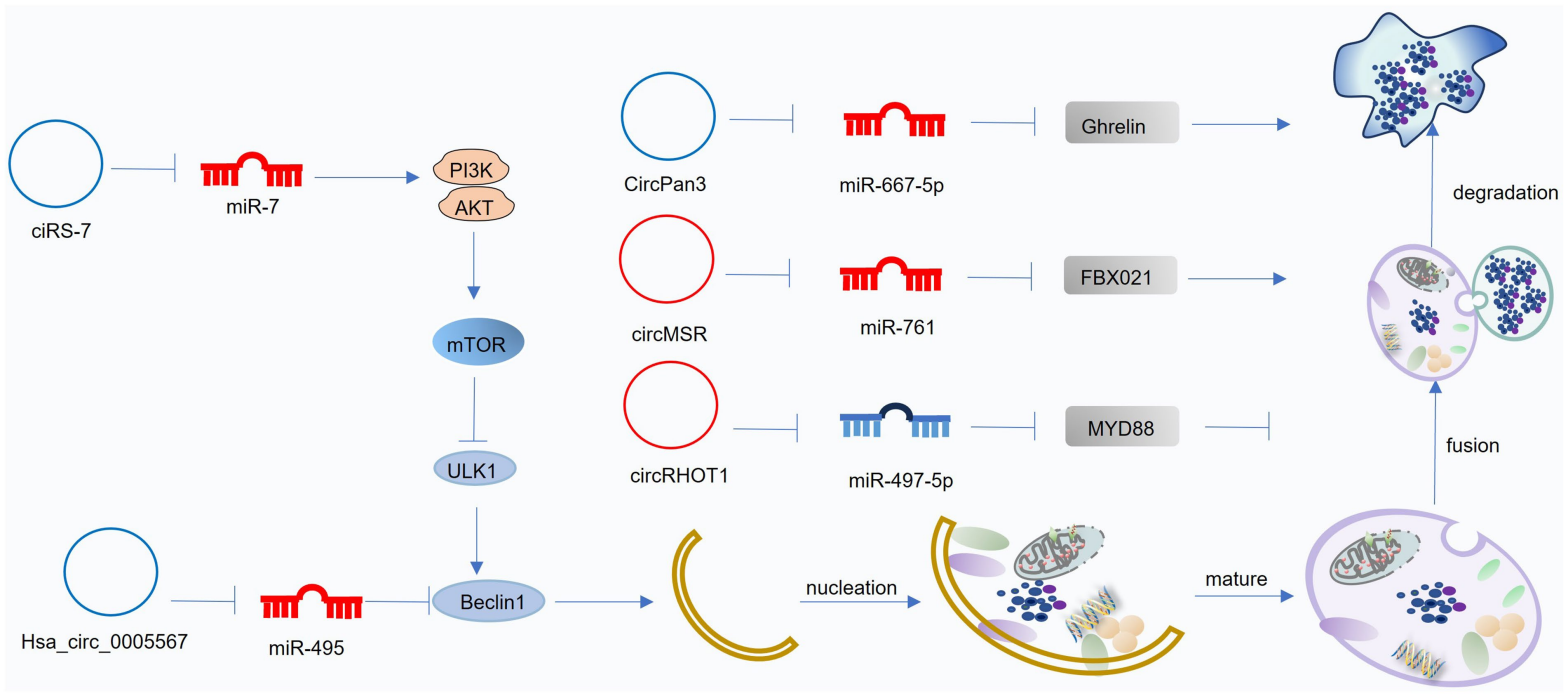
Mechanisms of circRNA-mediated regulation of autophagy in osteoarthritis (OA). CircRNAs modulate autophagy by acting as competing endogenous RNAs (ceRNAs) that influence downstream miRNA activity. Blue circRNAs indicate those enhancing autophagy and alleviating OA progression, whereas red circRNAs represent those suppressing autophagy and accelerating OA pathogenesis. This figure was generated and edited using Adobe Illustrator (Adobe Inc., United States).

CircRNAs are a class of endogenous, single-stranded, non-coding RNAs characterized by a covalently closed-loop structure formed through back-splicing. This unique structure confers enhanced stability to circRNAs compared to linear RNAs. CircRNAs exert their biological functions through various mechanisms. Firstly, CircRNAs can act as competing endogenous RNAs (ceRNAs), sequestering miRNAs and thereby preventing miRNA-mediated gene silencing. Secondly, CircRNAs can modulate gene transcription. Thirdly, CircRNAs can interact with RNA-binding proteins. Fourthly, Some circRNAs have been shown to possess protein-coding potential. Dysregulated circRNA expression patterns have been observed in OA (169). CircRNAs contribute to OA pathogenesis by modulating various cellular processes, including chondrocyte proliferation and apoptosis, cartilage erosion, and inflammation. Furthermore, circRNAs play a crucial role in regulating autophagy in OA, primarily through their ceRNA activity (Figure 6; Table 3).

**Table 3:** CircRNAs mediated autophagy in OA.

Model	CircRNA expression	Targeted MiRNAs	Targeting pathway	Effect on autophagy	Effect on cartilage or chondrocyte	Ref.
DMM induced OA	circFOXO3 ↓	/	FOXO3↑	Promote	apoptosis ↓ ECM synthesis ↑	(235)
Human OA cartilage, DMM induced OA	ciRS-7 ↓	miR-7	PI3K-Akt–mTOR↓	Promote	ECM synthesis ↑ ECM degradation ↓	(236)
IL-1β-inducd chondrocytes	hsa_circ_0005567 ↓	miR-495	ATG14↑	Promote	apoptosis ↓	(237)
DMM induced OA	CircPan3 ↓	miR-667–5p	Growth-releasing peptide↑	Promote	ADAMTS5, MMP13 ↓ Col2, ACAN ↑	(238)
IL-1β-inducd CHON-001 cells	hsa_circ_0037658 ↑	/	/	Inhibit	MMP13↑, ACANA ↓	(239)
Human OA cartilage	CircRNA-MSR ↑	miR-761	FBXO21↑	FBXO21↑	proliferation ↓	(240)
ACLT induced OA	circRHOT1 ↑	miR-142–5p	CCND1↑	Inhibit	apoptosis↑ proliferation ↓	(241)
IL-1β-induced human chondrocytes	circMELK ↑	miR-497–5p	Myeloid differentiation factor 88↑ NF-κB↑	Inhibit	inflammation↑ apoptosis ↑	(242)

#### 5.3.1 Positive circRNAs to suppress OA pathogenesis

Several circRNAs have been implicated in regulating autophagy and influencing OA pathogenesis. circFOXO3 acts as a sponge for its parental gene, FOXO3, inhibiting PI3K-Akt signaling and promoting autophagy. This, in turn, enhances collagen synthesis, suppresses apoptosis, and reduces ECM degradation in OA (170). CiRS-7 acts as a ceRNA for miR-7. Downregulation of CiRS-7 in OA leads to increased miR-7 activity, inhibiting autophagy and promoting cartilage degeneration. Conversely, miR-7 inhibition attenuates OA progression (171). hsa_circ_0005567 acts as a ceRNA for miR-495, promoting ATG14 expression and enhancing autophagy. circPan3 acts as a ceRNA for miR-667-5p, promoting the expression of growth-releasing peptide, which in turn activates autophagy and facilitates ECM repair (172).

#### 5.3.2 Negative circRNAs to promote OA pathogenesis

Several circRNAs contribute to OA pathogenesis by inhibiting autophagy. Upregulated in OA, hsa_circ_0037658 inhibits autophagy in chondrocytes. Silencing of hsa_circ_0037658 promotes autophagy, reduces MMP13 expression, and enhances collagen synthesis, mitigating OA progression (173). Upregulated in OA, hsa_circ_0037658 inhibits autophagy in chondrocytes. Silencing of hsa_circ_0037658 promotes autophagy, reduces MMP13 expression, and enhances collagen synthesis, mitigating OA progression (174). circRNA-MSR acts as a ceRNA for miR-761, leading to increased FBXO21 expression and impaired autophagy in OA (175). circRHOT1 acts as a ceRNA for miR-142-5p, promoting CCND1 expression and inhibiting autophagy. This contributes to chondrocyte apoptosis and OA progression (176). Upregulated in OA chondrocytes, circMELK acts as a ceRNA for miR-497-5p, promoting myeloid differentiation factor 88 (MyD88) expression and activating the NF-κB pathway. This leads to impaired autophagy, increased inflammation, and enhanced chondrocyte apoptosis (177). These findings demonstrate the diverse mechanisms by which circRNAs can regulate autophagy and contribute to OA pathogenesis.

This study categorized ncRNAs involved in OA autophagy into two groups: those that promote autophagy (positive regulators) and those that inhibit autophagy (negative regulators) (178). While the miRNA-autophagy network has been extensively explored, with miRNAs directly regulating various stages of autophagy, our understanding of the role of lncRNAs and circRNAs in this context is still evolving. Further research is needed to elucidate the intricate regulatory networks between these RNAs and autophagy in OA. Current OA models, such as DMM, ACTL, and inflammatory models induced by IL-1β or TNF-*α*, have identified numerous ncRNAs with altered expression levels (Tables 1–3). These ncRNAs may serve as valuable biomarkers for OA diagnosis and treatment. However, future studies should investigate ncRNA expression patterns in a broader range of OA models, including those associated with obesity, aging, and drug-induced injury, to gain a more comprehensive understanding of the ncRNA landscape in OA.

While the studies discussed above primarily focus on the role of ncRNAs in modulating basal or moderately elevated autophagy, it is crucial to acknowledge their potential involvement in regulating excessive autophagy, which can also contribute to OA pathogenesis (179). miR-27a targets PI3K, inhibiting the PI3K/Akt/mTOR pathway and promoting excessive autophagy, ultimately leading to chondrocyte death. In the SDF-1-induced OA model, miR-146a-5p targets CXCR4, inhibiting SDF-1/CXCR4 signaling and attenuating excessive autophagy. This lncRNA may contribute to the regulation of excessive autophagy, potentially through interactions with the miR-34a-5p/SYVN axis. Further research is warranted to identify additional ncRNAs that regulate excessive autophagy and to elucidate the underlying mechanisms contributing to OA pathogenesis.

## 6 Innovative approaches for targeting ncRNA-regulated autophagy in the treatment of OA

The extensive involvement of ncRNAs in regulating autophagy in OA suggests their significant potential as novel therapeutic targets. While research has primarily focused on ncRNAs that modulate basal or moderate levels of autophagy, the role of ncRNAs in regulating excessive autophagy, which can also contribute to OA pathogenesis, warrants further investigation.

### 6.1 Natural products

Current OA management primarily relies on pharmacological approaches, with NSAIDs, analgesics, and glucocorticosteroids constituting the mainstay of treatment. While these agents effectively alleviate OA symptoms, they are not without limitations. Long-term use of NSAIDs and glucocorticosteroids can lead to adverse effects on various organ systems, including the gastrointestinal and cardiovascular systems. Topical formulations, while minimizing systemic side effects, often exhibit poor penetration and require frequent application (180).

Given the increasing prevalence of drug-related side effects, natural products are emerging as promising alternatives for OA treatment. Their diverse chemical structures and generally low toxicity profile offer significant advantages (181). Many plant extracts, such as those derived from frankincense and Angelica dahurica, have demonstrated potent anti-inflammatory and antioxidant properties, effectively alleviating OA pain and dysfunction compared to conventional analgesics and glucosamine (182). Recent studies have highlighted the ability of natural products to modulate autophagy, a key cellular process implicated in OA pathogenesis. Propolis, a natural resin produced by bees, possesses a wide range of pharmacological properties, including antioxidant and anti-inflammatory effects. These beneficial effects are attributed to its rich polyphenol content, making it a promising candidate for natural anti-aging therapies (183). A recent study investigated the impact of ethanol extract of propolis (EEP) on miRNA expression in IL-1β-stimulated chondrocytes. Among five miRNAs known to regulate autophagy (miR-19a, miR-125b, miR-181a, miR-185, and miR-335), EEP treatment significantly downregulated miR-125b and upregulated miR-185, suggesting a potential role for these miRNAs in mediating the anti-inflammatory and autophagy-modulating effects of propolis in OA (184). Further investigation is needed to validate these findings and translate these preclinical observations into clinical applications for OA treatment.

### 6.2 Gene therapy

Gene therapy holds significant promise as a novel therapeutic approach for OA. The avascular nature of articular cartilage makes intra-articular injection an attractive route for delivering therapeutic nucleic acids, circumventing limitations associated with systemic drug delivery (185). Studies have demonstrated the therapeutic potential of intra-articularly injected miRNAs, such as miR-181a-5p and miR-34a-5p, in OA (186). However, the clinical translation of miRNA-based therapies faces challenges. Natural miRNAs exhibit limited stability within the joint environment, readily degrading due to enzymatic activity. Furthermore, efficient cellular uptake of these molecules remains a significant hurdle. Therefore, the development of effective gene delivery vectors is crucial for successful miRNA-based OA therapies. These vectors should protect miRNAs from degradation and facilitate their efficient delivery to target cells within the joint.

Nanoparticle-based delivery systems offer a promising approach for enhancing the efficacy and stability of miRNA therapeutics in OA. Chen et al. (187) demonstrated the efficacy of G5-AHP, a multifunctional polymeric nanoparticle, in delivering miR-224-5p to chondrocytes. Compared to conventional transfection methods, G5-AHP-mediated delivery significantly improved cellular uptake of miR-224-5p, leading to enhanced autophagy, reduced apoptosis, and improved ECM synthesis. Furthermore, intra-articular injection of G5-AHP-miR-224-5p nanoparticles in an OA mouse model significantly alleviated disease progression, as evidenced by reduced joint space narrowing, bone sclerosis, and synovial inflammation (188). These findings highlight the potential of nanoparticle-based delivery systems to overcome the limitations of traditional miRNA therapies and provide a promising avenue for the development of effective OA treatments.

Extracellular vesicles (EVs), including exosomes, microvesicles, and apoptotic bodies, are naturally occurring, membrane-bound vesicles released by various cell types (189). Compared to synthetic delivery systems like liposomes and polymeric nanoparticles, EVs offer several advantages, including high biocompatibility, low immunogenicity, and enhanced stability (190). Studies have shown that MSC-derived EVs exert therapeutic effects in OA by promoting cartilage regeneration. For example, MSC-derived exosomes from infrapatellar fat pads (IPFP-MSC-Exos) have been shown to reduce cartilage degradation and promote ECM synthesis in DMM-induced OA. Mechanistically, these exosomes deliver miR-100-5p, which inhibits mTOR signaling and promotes autophagy in chondrocytes (191). These EVs contain lncRNA NEAT1, which acts as a ceRNA for miR-122-5p. By downregulating miR-122-5p, NEAT1 increases the expression of its target gene, sestrin 2, activating the Nrf2 pathway and promoting autophagy in chondrocytes (192). These findings highlight the therapeutic potential of EVs in OA by delivering bioactive molecules, such as miRNAs and lncRNAs, to modulate autophagy and promote cartilage repair.

## 7 Conclusion and perspectives

Autophagy plays a crucial role in maintaining chondrocyte homeostasis. Under physiological conditions, autophagy serves as a protective mechanism, clearing cellular debris, mitigating oxidative stress, and promoting cellular survival. However, excessive autophagy can also contribute to chondrocyte death and OA progression. Therefore, therapeutic strategies targeting autophagy in OA should carefully consider the specific stage and context of the disease. Enhancing autophagy may be beneficial in situations where autophagy is impaired, such as in early stages of OA. Conversely, strategies to inhibit excessive autophagy may be necessary in later stages where autophagy contributes to chondrocyte death. A deeper understanding of the complex regulatory mechanisms governing autophagy in OA is crucial for developing effective therapeutic interventions.

Emerging evidence indicates that traditional Chinese medicine (TCM), including specific herbal components and manual therapies, can modulate autophagy-related pathways involved in the pathogenesis of OA. For instance, icariin, a flavonoid derived from Epimedium species, has been shown to alleviate OA in rat models by activating autophagy and suppressing the PI3K/AKT/mTOR signaling pathway, thereby enhancing chondrocyte viability and preserving cartilage integrity (193). In addition to herbal compounds, TCM-based manual therapy such as Tuina has been demonstrated *in vivo* to attenuate cartilage injury and inflammatory responses while regulating chondrocyte autophagy and apoptosis through the PI3K/AKT/mTOR pathway in experimental OA models (194). Beyond these interventions, accumulating evidence supports the autophagy-modulating effects of additional bioactive compounds derived from traditional Chinese herbal medicine. Oroxin B (OB), a flavonoid with documented anti-inflammatory properties, has been shown to alleviate chondrocyte injury by suppressing inflammatory signaling, inhibiting PI3K/AKT/mTOR activity, and promoting autophagy, suggesting its therapeutic potential in OA management (195). Similarly, Lou et al. demonstrated that glabridin (Gla), an isoprenylated isoflavone isolated from *Glycyrrhiza glabra*, effectively preserves extracellular matrix (ECM) homeostasis and maintains autophagic flux via modulation of the PI3K/AKT/FOXO3A signaling axis, highlighting a dual regulatory strategy for OA treatment (196). Moreover, Yang et al. reported that the Gubi Zhitong formula exerts protective effects against OA in both *in vitro* and *in vivo* models by regulating BNIP3L-mediated mitophagy, underscoring the importance of mitochondrial quality control in chondrocyte homeostasis (197).

Emerging evidence highlights a complex interplay between ncRNAs and autophagy in the pathogenesis of OA. While miRNAs have been extensively studied in this context, our understanding of the role of lncRNAs and circRNAs in regulating autophagy in OA remains limited. MiRNAs exert significant influence over autophagy by directly targeting key autophagy genes, including components of the mTOR pathway, ULK1, Beclin1, and ATG proteins, thereby modulating various stages of the autophagic process (198). LncRNAs and circRNAs primarily function as ceRNAs, indirectly influencing autophagy by regulating miRNA activity. However, the role of these ncRNAs in regulating both basal and excessive autophagy in OA requires further investigation. Furthermore, while the impact of altered autophagy levels on OA pathogenesis has been extensively studied, the specific role of ncRNAs in modulating excessive autophagy remains relatively unexplored (199). A comprehensive understanding of the complex interplay between ncRNAs and autophagy is crucial for developing novel therapeutic strategies for OA.

The therapeutic application of the ncRNA-autophagy axis in OA is still in its early stages. While promising, current research primarily focuses on preclinical models, with limited clinical translation. Compounds derived from natural sources offer potential therapeutic benefits due to their generally low toxicity profiles (200). However, challenges remain, including potential for systemic side effects and limitations in bioavailability. Gene therapy approaches, such as the use of gene delivery vectors to target specific ncRNAs, hold significant promise. However, optimizing delivery systems to ensure efficient and safe delivery of therapeutic agents to the target site within the joint remains a critical challenge. Key considerations include biocompatibility, biodegradability, and minimizing off-target effects. Further research is crucial to address these challenges and translate the promising preclinical findings into effective clinical therapies for OA (201).

OA pathogenesis is a complex process involving multiple factors, including chondrocyte senescence, inflammation, oxidative stress, and autophagy. While ncRNAs play a crucial role in regulating autophagy, they represent only one facet of the intricate network of factors contributing to OA (202). Furthermore, the interplay between ncRNAs and autophagy is multifaceted. A single study may not fully capture the complex interactions within this network. Therefore, a comprehensive understanding of OA pathogenesis, including the roles of various cellular processes and the intricate regulatory mechanisms involving ncRNAs, is crucial for the development of effective ncRNA-based therapies.
